# Chlamydial Infection: A Clinical and Public Health Perspective

**DOI:** 10.3201/eid2007.140490

**Published:** 2014-07

**Authors:** Gilbert Greub

**Affiliations:** University of Lausanne, Lausanne, Switzerland

**Keywords:** infectious diseases, gynecology, epidemiology, fertility, sexually transmitted infections, sexually transmitted diseases, STD, bacteria, Chlamydia

Chlamydial Infection: A Clinical and Public Health Perspective, a relatively short textbook, focuses on *Chlamydia trachomatis*, the most common bacterial agent of sexually transmitted infections. In the introduction, Black provides some definitions, a historical background, and a table summarizing the main clinical syndromes associated with urogenital serovars. Regrettably, this introduction does not mention the increasingly recognized role of urogenital chlamydial infections in miscarriage and infections caused by other members of the Chlamydiales order, such as *C. pneumoniae* and *Waddlia chondrophila*. Such mention would be especially useful because understanding the biology and evolution of *C. trachomatis* also relies partially on research performed on other chlamydiae, as is discussed nicely in the genomics chapter by T.E. Putman and D.D. Rockey.

A basic science chapter, “*Chlamydia trachomatis* Pathogenicity and Disease,” highlights the importance of *C. trachomatis* surface protein and host genetics in the immunopathogenesis of chlamydial infection. This chapter by Deborah Dean is enjoyable to read, highly informative, and represents an extended review with 292 references. The remaining 8 chapters emphasize epidemiology, clinical presentation, antimicrobial drug susceptibility, antibiotherapy, and vaccines. These chapters, written by field experts, provide precise practical recommendations about screening and diagnosis of, and treatment approaches to, urogenital chlamydial infection and about caring for sexual minority groups, such as men who have sex with men.

Overall, this textbook is an excellent reference for epidemiologists working on sexually transmitted infections, for clinicians interested in that field, and for doctoral students starting their research on *C. trachomatis*. However, given its relative conciseness, Black’s textbook is unlikely to meet the expectations of basic researchers working on the evolution, cell biology, and/or molecular microbiology of *Chlamydia*.

